# Supplementary material for: Sodium Houttuyfonate Prevents Seizures and Neuronal Cell Loss by Maintaining Glutamatergic System Stability in Male Rats with Kainic Acid-Induced Seizures
Source: Biomedicines. 2024 Jun 13;12(6):1312. doi: 10.3390/biomedicines12061312 (PMC11202147; doi:10.3390/biomedicines12061312)
Supplement: Supplementary file 1 [file biomedicines-12-01312-s001.zip › biomedicines-3013102-supplementary.pdf]

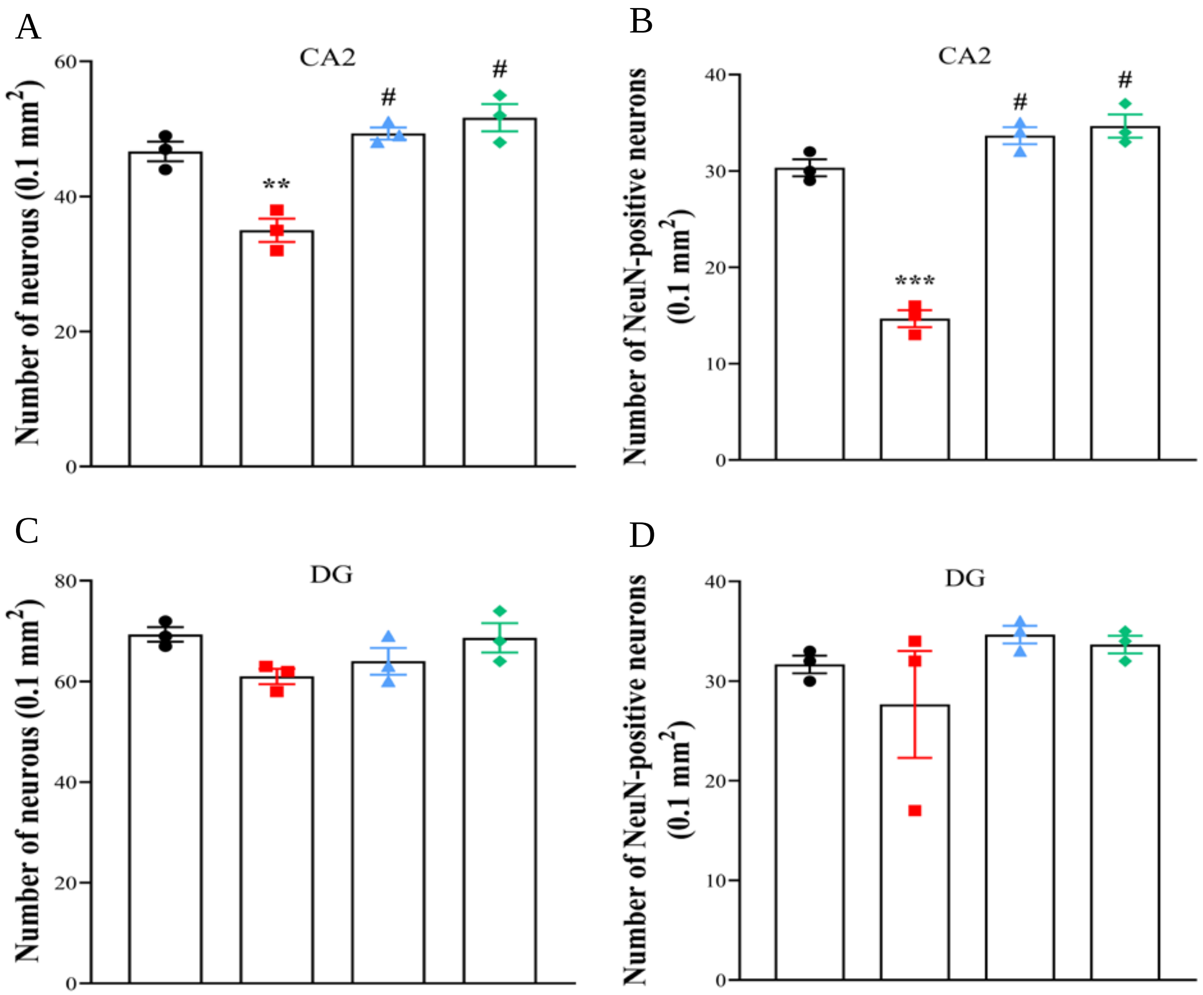

Figure S1. Quantitative data (B, C) for Cresyl violet staining (A, C) and NeuN staining (B, D). N = 3 animals per group. ** $p < 0.001$, *** $p < 0.0001$ compared to the control group; # $p < 0.0001$ compared to the KA group.
